# Characteristics of ‘mega’ peer-reviewers

**DOI:** 10.1186/s41073-022-00121-1

**Published:** 2022-02-21

**Authors:** Danielle B. Rice, Ba’ Pham, Justin Presseau, Andrea C. Tricco, David Moher

**Affiliations:** 1 Centre for Journalology, Clinical Epidemiology Program, The Ottawa Hospital Research Institute, Ottawa, Canada; 2 Department of Psychology, Faculty of Science, McGill University, Montreal, Canada; 3 Institute of Health Policy, Management and Evaluation, University of Toronto, Toronto, Canada; 4 Clinical Epidemiology Program, The Ottawa Hospital Research Institute, Ottawa, Canada; 5 Faculty of Medicine, School of Epidemiology and Public Health, University of Ottawa, Ottawa, Canada; 6 Faculty of Social Sciences, School of Psychology, University of Ottawa, Ottawa, Canada; 7 Knowledge Translation Program, Unity Health Toronto, Li Ka Shing Knowledge Institute, St. Michael’s Hospital, Toronto, Canada; 8 Epidemiology Division and Institute for Health Policy, Management, and Evaluation, Dalla Lana School of Public Health, University of Toronto, Toronto, Canada; 9 Queen’s Collaboration for Health Care Quality Joanna Briggs Institute Centre of Excellence, Queen’s University, Kingston, Canada

**Keywords:** Peer review, Characteristics of reviewers, Publons

## Abstract

**Background:**

The demand for peer reviewers is often perceived as disproportionate to the supply and availability of reviewers. Considering characteristics associated with peer review behaviour can allow for the development of solutions to manage the growing demand for peer reviewers. The objective of this research was to compare characteristics among two groups of reviewers registered in Publons.

**Methods:**

A descriptive cross-sectional study design was used to compare characteristics between (1) individuals completing at least 100 peer reviews (‘mega peer reviewers’) from January 2018 to December 2018 as and (2) a control group of peer reviewers completing between 1 and 18 peer reviews over the same time period. Data was provided by Publons, which offers a repository of peer reviewer activities in addition to tracking peer reviewer publications and research metrics. Mann Whitney tests and chi-square tests were conducted comparing characteristics (e.g., number of publications, number of citations, word count of peer review) of mega peer reviewers to the control group of reviewers.

**Results:**

A total of 1596 peer reviewers had data provided by Publons. A total of 396 M peer reviewers and a random sample of 1200 control group reviewers were included. A greater proportion of mega peer reviews were male (92%) as compared to the control reviewers (70% male). Mega peer reviewers demonstrated a significantly greater average number of total publications, citations, receipt of Publons awards, and a higher average h index as compared to the control group of reviewers (all *p* < .001). We found no statistically significant differences in the number of words between the groups (*p* > .428).

**Conclusions:**

Mega peer reviewers registered in the Publons database also had a higher number of publications and citations as compared to a control group of reviewers. Additional research that considers motivations associated with peer review behaviour should be conducted to help inform peer reviewing activity.

Peer review involves a manuscript undergoing evaluation of research by, for example, experts or early career researchers in the same field, individuals with lived experience, and policy advisors [1]. When peer reviewers are aware and in agreement with the expectations and responsibilities of reviewing an article, and editors incorporate feedback in a timely manner, peer review has the potential to result in valuable feedback for the authors and improve the quality and usability of research findings [2].

The sustainability of peer review relies on the availability and expertise of peer reviewers. Obtaining peer reviews that are high quality is difficult for many journal editors [3–8]. An inaugural Global State of Peer Review report, developed by Publons in collaboration with the Web of Science group (both owned by Clarivate Analytics), reported on (1) characteristics of peer reviewers, (2) efficiencies of the peer-review process, (3) quality of peer review, and (4) future considerations for peer review. Importantly, this report described that “demand for peer review is increasing with reviewers becoming less responsive to review invitations” [2]. Certain characteristics were found to be associated with the completion of peer review activities. For example, variability between regions for peer reviewer activity exists, with individuals from the USA and China contributing the greatest number of peer review [2]. Regional variability in the incentives structure has been suggested as one factor that may partially account for these differences. The Publons’ 2018 Global Review Survey included over 11,800 researchers and found overwhelming agreement (85% of participants) that greater recognition and formalized incentives for peer review would increase willingness to serve as a peer reviewer and would positively impact the efficiency of the peer-review process [2]. Traditional rewards exist, such as journal subscriptions, discounts for open accessing publishing, and acknowledgements through public “thank you” lists. However, these rewards are inconsistently applied among journals and do not meet the preferred rewards and incentives (e.g., waiver of publication fees) and recognition (e.g., incorporated as part of the evaluation criteria for funding applications) being sought by researchers [2, 9–11]. Further, findings from the Global State of Peer Review report includes a summary of trends found among a large sample of peer reviewers and the current strain on the peer review system. There remain substantial gaps in the understanding of characteristics of individuals that agree to serve as peer reviewers and the quality of peer reviews [2].

Publons offers a repository where peer reviewers can document their peer review activities in addition to tracking publications and research metrics [12]. Anecdotally, we have observed some researchers that are highly active in peer reviewing (i.e., individuals completing at least 100 peer reviews, annually – we refer to these individuals as ‘mega peer reviewers’) on the Publons website. A recent study also highlighted the unequal distribution of peer-reviewing tasks among small groups of researchers [13]. Identifying characteristics associated with mega peer reviewers may be a first step in developing strategies for keeping pace with the growing demand for peer review tasks. For instance, this could provide editors with information about researchers that have experience handling more bandwidth of peer reviews, a continual problem for editors. As such, the objective of this research was to compare two groups of reviewers, specifically, individuals who were highly active peer reviewers in a given year as compared to a control group of peer-reviewers. Given the lack of research on mega peer reviewers, this was an exploratory project, and we did not form hypotheses.

## Methods

The protocol for this study was registered within the Open Science Framework database (https://osf.io/vxdhf/?view_only=313fd05399664b94bc7a9042aa225be3) before data collection began. This was a descriptive cross-sectional study that retrospectively examined factors associated with mega peer reviewers compared to a control group of peer reviewers. Mega reviewers were defined as individuals that completed peer reviews for 100 or more unique articles from January 2018 to December 2018. All aspects of this study were reported in accordance with the original Strengthening (STROBE) reporting guidelines to facilitate the complete and transparent reporting of this work [14].

### Cross sectional data

#### Participants

We gathered information from the Publons database. Publons tracks and publicizes peer-reviewer activities for individuals that create an account and connect their research activities to their profile. Individuals can download their peer-review, author, and editor metrics and this information can also be made public. Using the Publons database, two groups of individuals were of interest for this study, including [1] mega peer reviewers: all individuals that completed peer reviews for 100 or more unique articles from January 2018 to December 2018, inclusive (i.e., individuals completing approximately two peer reviews every week) and [2] a control group of individuals completing at least one peer review and less than 18 peer reviews over the same time period (i.e., individuals completing up to 1 peer review every 3 weeks). A random sample of controls were selected from Publons database using Pandas sample method (https://pandas.pydata.org/pandas-docs/stable/reference/api/pandas.DataFrame.sample.htmal).

#### Data collection

A data scientist from Publons exported the following variables into a csv Microsoft excel file: peer reviewer characteristics [i.e., name, publons ID, institution, country of institution, number of publications based on Publons data, publications in Web of Science, publications in 2018, citations in 2018, total number of citations, h-index, presence of a Publons reviewer award (top 1% of reviewers in 22 research areas, top quality reviews based on editor rated evaluations)], review characteristics based on Publons data (i.e., number of unique manuscripts peer-reviewed in 2018, number of unique manuscripts reviewed each month, number of words per review). Sex was not available on Publons. As such, sex was estimated by using the Genderize data base, which uses data collected from countries to assess the probability of the sex being associated with a given name (https://genderize.io/). For any sex that could not be estimated with more than 80% certainty, this was marked as missing data.

#### Sample size calculation

In our registered protocol, the sample size calculation was incorrectly determined (see Appendix 1). We conducted the study using data from all mega-reviewers and a random sample of 1200 reviewers with 1 to 18 conducted reviews per year.

#### Data analysis

Primary data analysis calculated descriptive characteristics of both samples of reviewers. The secondary data analysis involved conducting a logistic regression to compare the mega peer reviewer characteristics to the control group, treating mega-reviewing as a binary outcome. Given the exploratory nature of this study, the association between peer reviewer characteristics (i.e., sex, country of institution, number of publications in Web of Science, publications in 2018, citations in 2018, total number of citations, h-index, presence of Publons reviewer award) and review characteristics (i.e., number of words per review) (independent variables) were compared in the two groups of reviewers (mega-reviewers and control group of reviews) within the regression model. Prior to conducting regression analyses, preliminary tests were performed to determine the appropriateness of analyses based on any violations of regression assumptions. Tests of multicollinearity were conducted, and independent variable tolerance values were reviewed. Four variables did not reach the recommended cut-off values for collinearity statistics [15], suggesting that variables were highly correlated with one another [tolerance values less than 0.1 for publications based on Publons (0.04), publications based on Web of Science (0.04), citations in 2018 (0.06), total citations (0.06). Distribution of variables was tested through inspection of the normal probability plots of the residuals and independence of residuals on the basis of the standardised residual and scatter plot inspections. The variables included in analyses were not normally distributed. As such, Mann-Whitney u tests for continuous data and chi-square analyses for categorical data were used. Analyses were conducted using SPSS version 27.0, and statistical tests were two sided with a significance value of *P* < .05.

## Results

### Deviations from protocol

Conducting a survey with mega reviewers and control reviewers to better understand what drives peer reviewing behaviour was a planned part of the current project. This survey has not yet been conducted due to reduced availability of the first author. The average word count of reviewers at the same institutions as the mega reviewers and control reviewers was removed from the included variables as this variable is a characteristic of the institution rather than the individual reviewers.

### Demographic characteristics

A total of 396 M reviewers that completed > 100 peer reviews during 2018 were included. A random sample of 1200 control group reviewers completing 18 peer reviews over the same time period. For reviewers that could have their sex estimated with at least 80% certainty (*n* = 1315), 92% of mega peer reviewers were male, as compared to 70% of reviewers that were male in the control group. Characteristics of mega peer reviewers and the control group are included in Tables 1 and 2.

**Table 1:** T-Test Results Comparing Characteristics of Mega Reviewers and Control^a^ Reviewers

Characteristics	Control Reviewers Median (Interquartile Range)	Mega Reviewers Median (Interquartile Range)
**Publications**^**b**^	0 (0–28)^d^	77 (19–153)^d^
**Publications**^**c**^	0 (0–23)^d^	57 (12–129) ^d^
**Publications 2018**	0 (0–1)^d^	5 (0–12) ^d^
**Citation Count 2018**	44 (16–118)^d^	217 (49–580)^d^
**H Index**	9 (5–16)^d^	20 (9–34)^d^
**Citation Count Total**	300 (94–1093)^d^	1244 (255–3491)^d^
**Articles Reviewed 2018**	3 (2–5)^d^	112 (94–153)^d^
**Word Count**	256 (78–465)	228 (163–328)

^a^ = reviewers with 1 to 18 completed peer reviews, ^b^based on Publons data, ^c^Based on Web of Science data, ^d^between group difference of *p* < .001

**Table 2:** Chi-Square Analyses Results Comparing Characteristics of Mega Reviewers and Control Reviewers

Characteristics	Control (% of total)	Mega Reviewers (% of total)	***P***-Value
**Continent**			
North America	269 (25.8)	70 (18.9)	.001
Australia	63 (6.0)	16 (4.3)	
Europe	428 (41.1)	136 (36.8)	
Asia	222 (21.3)	122 (33.0)	
South America	32 (3.1)	3 (0.8)	
Africa	28 (2.7)	23 (6.2)	
**Sex**			
Male	690 (57.5)	296 (74.7)	.001
Female	304 (25.3)	25 (6.3)	
Missing^a^	206 (17.2)	75 (18.9)	
**Publons Awards**			
No	1193 (99.4)	47 (11.9)	.001
Yes	7 (0.6)	349 (88.1)	

^a^Sex that could not be estimated with more than 80% certainty using https://genderize.io/

### Mann-Whitney and Chi-Square analyses

A series of Mann-Whitney u tests were conducted comparing mega peer reviewers to the control group of reviewers (see Table 1). Mega peer reviewers had a significantly greater median number of publications (total), publications in 2018, citations (total), citations in 2018, and a significantly higher average h index as compared to the control group of reviewers (all *p* < .05). No statistically significant differences in the medians were found when comparing for the number of words in peer reviews between groups (*p* > .05) (see Table 1).

The continent of reviewers significantly differed. The majority of mega peer reviewers were from Asia (33%), Europe (37%), and North America (19%). Among the control group of peer reviewers, 41% were from Europe, 26% from North America, and 21% from Asia. The remaining reviewers were from Australia (mega peer reviewers = 4%; control peer reviewers = 6%), South America (mega peer reviewers = 1%; control peer reviewers = 3%), and Africa (mega peer reviewers = 6%; control peer reviewers = 3%). Publons awards were significantly more present among mega peer reviewers with 88% of mega peer reviewers having received an award from Publons as compared to less than 1 % of the control group reviewers (see Table 2).

## Discussion

Our study has found that reviewers that had peer reviewed at least 100 papers within a 12 month time-frame had more publications and citations in total and within a one-year time frame, a higher h index, and received more Publons awards as compared to the control group of reviewers that had reviewed between 1 to 18 reviews as indexed in the Publons database. Our study was not designed to explore the reasons for these differences. For example, mega reviewers could be invited to review more often by editors, or they could be receiving payment for peer reviewing, however, the findings are in line with previously conducted research about peer reviewers’ academic impact [16].

Many mega peer reviewers were from Asia or Europe, whereas over half of the control group was from Europe or North America, with fewer from Asia. The geographical regions of control group reviewers more closely aligned to findings from Publons’ 2018 Global Review Survey, where a large portion of reviewers were from Europe or North America [2]. Notably, mega peer reviewers were overwhelmingly male. This finding, however, is complex. It likely is at least partly a reflection of many female academics managing multiple responsibilities at work and at home, resulting in little extra bandwidth to take on a peer review load that is as substantial as mega peer reviewers [17, 18]. Conducting a survey with mega reviewers and control reviewers to better understand what drives reviewer behaviour was planned, however, this has not been completed due to reduced availability of the first author. Our study provides information on the characteristics of reviewers that may be willing to complete a large number of peer reviewing activities. Our results suggest that mega-peer reviewers may be more established [e.g., more citations; high h-index] than non-mega peer reviewers. Editors struggling to find peer reviewers for articles may want to consider inviting mega-peer reviewers in their field of study.

Our findings demonstrate a substantial time commitment among mega peer reviewers in completing a task that is often perceived as burdensome [13]. The estimated cost of peer reviewing totals more than 100 million hours in 2020 alone [19]. Mega peer reviewer’s altruism and dedication to peer reviewing should be acknowledged. The total number of articles reviewed in 2018 by mega peer reviewers was over 54,000. These articles were peer reviewed by 396 individuals, and this represents 11 times more peer reviews than the 1200 individuals in the control group completed. When considering the number of peer reviews completed by mega reviewers, it is possible that the level of detail provided to authors is less comprehensive compared to control peer reviewers, however, no significant difference was found for this variable. Limited length of peer reviews may be common regardless of the number of reviews completed each year. A recent study evaluated over 1400 sets of reviewer comments and found that 19% of reviews provided superficial comments and very little helpful guidance to authors [5]. Conducting a similar study focused on reviews completed by mega reviewers is important to better understand the impact of completing a substantial number of peer reviewing activities.

Both categories of peer reviewers provide approximately two thirds of a page of peer reviewer feedback per article with mega peer reviewers using fewer words. Both groups of reviewers in this study provide less than a page of text for a review which may be inadequate for providing constructive feedback for an entire manuscript. Assuming a brief opening paragraph to precis the research paper under peer review (i.e., providing the authors with a measure of face validity about the peer reviewers understanding of the research report), followed by optimal reviewing with the help of a reporting guideline [18], along with any specific journal reviewing guidance, it is not clear that all of this information can be conveyed in so few words. Additionally, peer reviews are most helpful to authors when they are evidence based [20], often necessitating citing making the word length even longer. It is possible, however, that rather than intending to be helpful, some mega peer reviewers are trying to influence what is published in their field [21]. For instance, they may complete many reviews with a decision of “reject” while providing little feedback for authors.

Publons Academy modules provide relevant knowledge to all peer reviewers who are trainees, which could influence the quality and completeness of peer reviews. The need to prioritize depth over the number of completed peer reviews may need to be further emphasized in these modules when training reviewers. This emphasis on depth may also be relevant for incentive structures. Peer reviewing is often absent from incentive structures within academia [13], however, certain institutions have started to incorporate peer reviewing activities into career advancement [11]. It is currently unknown if mega peer reviewers are rewarded at their institutions for peer reviewing and if there are other incentives contributing to mega peer reviewers behaviour. Future research that identifies qualitative and quantitative barriers and enablers associated with peer-reviewing behavior can provide a basis for keeping pace with the growing demand for peer reviewers. It can also identify facilitators and barriers to producing high-quality peer reviews. Conducting a survey with mega reviewers and control reviewers to better understand the current study findings was a planned part of this work but has not yet been completed. To inform change, also surveying editors and associated editors would provide a more thorough understanding of how mega peer reviewers receive ongoing journal request for peer reviews and understanding why editors may frequently invite specific reviewers.

There are limitations that should be considered when interpreting our results. First, the data used was collected from researchers that have an account through Publons database which may result in a selection bias. Second, the data collected for this work was received from Publons, limiting the variables that were available. For example, the sex of peer reviewers is not collected within the Publons database. As such, we estimated gender using an online algorithm that has been used in previous studies [22]. The accuracy of estimating gender has been previously studied and can result in a bias towards English names; it also reduces gender to a binary variable [23]. Our findings of the number of reviewers that are male and female should be considered with this understanding and should not be overinterpreted. Third, assessing the quality of peer-reviews was not possible based on available data. The recommendations provided by reviewers was also not available. This precludes an objective interpretation of the number of words provided for each review within our study, as previous research has found that the number of words provided for a review is associated with the recommendation of the reviewer (i.e., accept, revise, reject) [24] and field of the article [25]. Relatedly, our sample size calculation assumed that average word count was similar across disciplines. While it is possible that pre-existing differences between groups would balance out, this assumption may conceal differences between controls and mega peer reviewers. Finally, considering geographical variability in peer reviewing activities was limited, due to few reviewers in either group being located in Australia, South America, or Africa. Finally, since we initiated this study, Clarivate Analytics purchased Publons. The peer review training offered by Publons Academy now exists as the Web of Science academy. [https://clarivate.com/webofsciencegroup/solutions/web-of-science-academy/].

## Conclusions

The demand for peer reviewers continues to increase. Peer review can result in valuable feedback for the authors and improve the quality and usability of research findings. However, obtaining a high quality peer review is difficult for many journals. The current research found that mega peer reviewers complete a substantive number of peer review activities each year and demonstrate significantly different characteristics than a group of control peer reviewers. These characteristics are important to understand in order to increase the availability and usability of peer reviewers. Future research that identifies the factors associated with peer-review behaviour should be conducted to help inform designing strategies and interventions to facilitate system- and individual- level changes in peer reviewing activity.

## Data Availability

The dataset supporting the conclusions of this article is available at https://osf.io/6awzr/?view_only=eeb8ccc0c3a7468095f0b7ef67b71508

## Appendix 1

Estimate sample size of the random sample of the control group.

In our registered protocol, the sample size calculation was incorrectly determined. The mega peer-reviewers sample size was based on the number of peer-reviewers on the Publons website that met our inclusion criteria (i.e., greater than 100 peer-reviews 2018). For the control group, a sample size calculation based on the total number of reviewers that met the control group requirements (i.e., completing at least one review and less than 18 peer-reviews in 2018) was conducted using the standard deviation of the average word count which was estimated using preliminary data from Publons. A sample size calculation was conducted in R package (pwr) for a two sample t-test comparing mega peer reviewers and the control group. The pooled standard deviation was calculated, and a minimum sample size of 1167 was estimated (see Appendix 1). To determine the number of peer reviewers needed for the control group, a 1:1 random sample was selected and the standard deviation of the average word count of review reports was determined. This sample size calculation assumes that the average word count of reviews is a uniformly distributed variable, however, this variable may not be uniformly distributed across reviews given known differences in the average number of words used across disciplines. This assumption may mask relevant differences in word count by discipline.

**Data**

**Table:**

Average word count	Mega reviewers (*n* = 396)	1:1 Matched Control (*n* = 396)
Median	228	254
Mean	272	326
Standard deviation	220	333
Standard error of the mean	11	17

**Assumptions**

- With a sample size of > 800 reviewers, the t-test is appropriate to compare the two groups of reviewers.
- With the current group of controls (*n* = 396), the mean difference in the average word count between the groups of mega reviewers and controls is 54 words, or approximately three sentences (average 60 words), assuming that the average word count per sentence is 20 words.
- With the current group of controls, the pooled standard deviation is 282 words (or slightly more than a half of a page (average 500 words per page).
- Given that the size of the “population” of 186,184 controls, we guestimate that the standard deviation of the control may increase from 333 words (with 396 controls) to 500 words (with 186,184 controls).
- Sample size is estimated using R package “pwr” for t-test (below).

**Recommendation:** Random sample of 1200 controls.

**Calculation of the pooled standard deviation**

**Table:**

Mega reviewers		Control		Pooled	Mega	Control	
n1	sd1	n2	sd2	SD	mean1	mean2	Diff
396	220	396	333	282	272	326	54
396	220	800	500	428			
396	220	1200	500	447			

# Sample size calculation.

power.t.test(power = 0.9,delta = 60,sd = 428,type = “two.sample”).

> library(pwr) # https://cran.r-project.org/web/packages/pwr/vignettes/pwr-vignette.html

> power.t.test(power = 0.9,delta = 60,sd = 428,type = “two.sample”).

Two-sample t test power calculation.

*n* = 1070.29.

delta = 60.

sd = 428.

sig.level = 0.05.

power = 0.9.

alternative = two.sided.

NOTE: n is number in *each* group

> power.t.test(power = 0.9,delta = 60,sd = 447,type = “two.sample”).

Two-sample t test power calculation.

*n* = 1167.338 ← This matches with our assumption in Table 2 of 1200 reviewers.

delta = 60.

sd = 447.

sig.level = 0.05.

power = 0.9.

alternative = two.sided.

**NOTE**: *n* is number in *each* group

## Abbreviations

STROBE: The Strengthening the Reporting of Observational Studies in Epidemiology
SD: Standard deviation

## References

[1] Elsevier (2020). What is peer review.

[2] Publons. 2018 Global State of Peer Review 2018. Available from: https://publons.com/community/gspr#open-elq-form-slider-DLGSPR. Accessed December 20, 2020.

[3] Beaumont LJ (2019). Peer reviewers need a code of conduct too. Nature..

[4] Bohannon J (2013). Who's afraid of peer review. Science..

[5] Gerwing TG, Gerwing AMA, Avery-Gomm S, Choi C-Y, Clements JC, Rash JA (2020). Quantifying professionalism in peer review. Res Integr Peer Rev..

[6] Gerwing TG, Gerwing AMA, Choi C-Y, Avery-Gomm S, Clements JC, Rash JA (2021). Reevaluation of solutions to the problem of unprofessionalism in peer review. Res Integr Peer Rev.

[7] Hyland K, Jiang FK (2020). ‘‘this work is antithetical to the spirit of research”: an anatomy of harsh peer reviews. J Engl Acad Purp.

[8] Mulligan A, Hall L, Raphael E (2013). Peer review in a changing world: an international study measuring the attitudes of researchers. J Assoc Inf Sci Technol.

[9] Tennant JP, Dugan JM, Graziotin D (2017). A multi-disciplinary perspective on emergent and future innovations in peer review. F1000Research.

[10] Nicholson J, Alperin JP. A brief survey on peer review in scholarly communication. The Winnower. 2016:1–8.

[11] Moher D, Bouter L, Kleinert S, Glasziou P, Sham MH, Barbour V, Coriat AM, Foeger N, Dirnagl U (2020). The Hong Kong principles for assessing researchers: fostering research integrity. PLoS Biol.

[12] Publons. 2020. Available from: https://publons.com/about/mission. Accessed March 7, 2020.

[13] Severin A, Chataway J. Overburdening of peer reviewers. A multi-disciplinary and multi-stakeholder perspective on causes, effects and potential policy implications. bioRxiv. 2021:1–19. 10.1101/2021.01.14.426539.

[14] Von Elm E, Altman DG, Egger M (2008). The strengthening the reporting of observational studies in epidemiology (STROBE) statement: guidelines for reporting observational studies. J Clin Epidemiol.

[15] Tabachnick B, Fidell LS. Using multivariate statistics: international edition. Pearson. 2013.

[16] Lei Y. Is a journal's ranking related to the reviewer's academic impact?(an empirical study based on Publons). Learned Publishing. 2021:1–14. 10.1002/leap.1431.

[17] Matulevicius SA, Kho KA, Reisch J, Yin H (2021). Academic medicine faculty perceptions of work-life balance before and since the COVID-19 pandemic. JAMA Netw Open.

[18] Aldossari M, Chaudhry S (2021). Women and burnout in the context of a pandemic. Gender, Work & Organization.

[19] Aczel B, Szaszi B, Holcombe AO (2021). A billion-dollar donation: estimating the cost of researchers’ time spent on peer review. Res Integr Peer Rev..

[20] Network EQUATOR (2021). Enhancing the QUAlity and transparency of health research.

[21] Moher D, Jadad AR. How to peer review a manuscript. Peer Rev Health Sci BMJ Books, London. 2003:183–90.

[22] Kelly J, Sadeghieh T, Adeli K (2014). Peer review in scientific publications: benefits, critiques, & a survival guide. EJIFCC..

[23] Santamaría L, Mihaljević H (2018). Comparison and benchmark of name-to-gender inference services. PeerJ Comput Sci.

[24] Casnici N, Grimaldo F, Gilbert N, Squazzoni F (2017). Attitudes of referees in a multidisciplinary journal: an empirical analysis. J Assoc Inf Sci Technol.

[25] Horbach SP, Halffman W (2018). The changing forms and expectations of peer review. Res Integr Peer Rev.

