# Correction: Characteristics of ‘mega’ peer-reviewers

**DOI:** 10.1186/s41073-022-00124-y

**Published:** 2022-07-13

**Authors:** Danielle B. Rice, Ba’ Pham, Justin Presseau, Andrea C. Tricco, David Moher

**Affiliations:** 1 Centre for Journalology, Clinical Epidemiology Program, The Ottawa Hospital Research Institute, Ottawa, Canada; 2 Department of Psychology, Faculty of Science, McGill University, Montreal, Canada; 3 Institute of Health Policy, Management and Evaluation, University of Toronto, Toronto, Canada; 4 Clinical Epidemiology Program, The Ottawa Hospital Research Institute, Ottawa, Canada; 5 Faculty of Medicine, School of Epidemiology and Public Health, University of Ottawa, Ottawa, Canada; 6 Faculty of Social Sciences, School of Psychology, University of Ottawa, Ottawa, Canada; 7 Knowledge Translation Program, Unity Health Toronto, Li Ka Shing Knowledge Institute, St. Michael’s Hospital, Toronto, Canada; 8 Epidemiology Division and Institute for Health Policy, Management, and Evaluation, Dalla Lana School of Public Health, University of Toronto, Toronto, Canada; 9 Queen’s Collaboration for Health Care Quality Joanna Briggs Institute Centre of Excellence, Queen’s University, Kingston, Canada

**Correction to: Res Integr Peer Rev 7, 1 (2022)**

**https://doi.org/10.1186/s41073-022-00121-1**

Following publication of the original article [1], the authors identified an error in the ‘Results’, both in the Abstract and in the main text: it incorrectly stated that ‘a greater proportion of mega peer reviews were male (92%) as compared to the control reviewers (70% male)’, instead of 74% vs 58% as listed in the table.

In addition, ‘Web of Science’ needed to be changed to ‘Clarivate’ in the main text and the ‘Acknowledgements’ section.

The original article [1] has been corrected.
